# *Talaromyces amestolkiae* Infection in an AIDS Patient with Cryptococcal Meningitis

**DOI:** 10.3390/jof9090932

**Published:** 2023-09-15

**Authors:** Li-An Wang, Yu-Chuan Chuang, Ting-Kuang Yeh, Kuan-Pei Lin, Chi-Jan Lin, Po-Yu Liu

**Affiliations:** 1 Department of Internal Medicine, Taichung Veterans General Hospital, Taichung 40705, Taiwan; 2 Division of Infectious Diseases, Department of Internal Medicine, Taichung Veterans General Hospital, Taichung 40705, Taiwan; 3 Genomic Center for Infectious Diseases, Taichung Veterans General Hospital, Taichung 40706, Taiwan; 4 Institute of Molecular Biology, National Chung Hsing University, Taichung 40227, Taiwan; 5 Department of Post-Baccalaureate Medicine, College of Medicine, National Chung Hsing University, Taichung 40227, Taiwan

**Keywords:** lymphadenopathy, co-infection, immunocompromised, talaromycosis, internal transcribed spacer, beta-tubulin

## Abstract

Concurrent infections caused by multiple fungal pathogens in immunocompromised patients can pose diagnostic and treatment challenges. Here, we presented the first reported case in Taiwan of an AIDS patient who had concurrent infection with *Cryptococcus neoformans* meningitis and *Talaromyces amestolkiae* lymphadenopathy. The patient presented with an enlarged inguinal lymph node and was diagnosed with *T. amestolkiae* lymphadenitis. The species *T. amestolkiae* was identified using DNA sequencing, which had the capability of differentiating it from other *Talaromyces* species. The patient was discharged from the hospital following treatment with amphotericin B and subsequent administration of voriconazole. This case highlights the importance of maintaining a suspicion of co-infections and utilizing appropriate diagnostic tools, such as DNA sequencing, to identify possible pathogens. Further studies are needed to determine the optimal treatment for *T. amestolkiae* and other co-infecting fungal pathogens.

## 1. Introduction

The genus *Talaromyces* was first described by Benjamin in 1955 based on phylogenetic information, which comprised several perfect stages of *Penicillium* species [1]. It was later re-classified by Yimaz et al. based on morphological, molecular, and physiological characters, and consisted of over 80 species, including *T. marneffei*, which was frequently found in immunocompromised patients [2]. Other *Talaromyces* species, including *Talaromyces amestolkiae*, *Talaromyces purpurogenus*, and *Talaromyces piceus*, have also been reported to cause invasive fungal infections [3].

Yilmaz et al. first described *T. amestolkiae* in 2012. *T. amestolkiae* was mostly isolated from the environment, including indoor dust, indoor air, soil, and wheat. It was also found in the sputum and lungs of cystic fibrosis patients [4]. Although *T. amestolkiae* has been shown to be a potential pathogen in humans, the current diagnostic tools are insufficient. 

While there are several diagnostics tools available, including microbial cultures, immunological techniques, microscopy, and PCR assays, certain fungal infections remain misdiagnosed or underdiagnosed. Differentiating among various *Talaromyces* species using conventional clinical microbiology tools is difficult, as most species are red pigment-producing and exhibit similar features under the microscope. Therefore, the development of more specific and sensitive diagnostic techniques for *Talaromyces* species, including *T. amestolkiae*, is essential for accurate identification and timely treatment [3]. However, currently, there is limited information in the literature regarding the *Talaromyces* species, other than *T. marneffei*.

This report discussed the first AIDS case with cryptococcal meningitis and lymphadenopathy due to a *T. amestolkiae* infection. This report covered details regarding clinical characteristics and treatments of *T. amestolkiae* infection. Molecular and morphological comparisons between *T. amestolkiae* and *T. marneffei* were carried out to facilitate better differentiation between the two species. 

## 2. Case Report

A 31-year-old male patient with AIDS was admitted to the hospital with headache, diplopia, bilateral inguinal lymphadenopathy, and several erythematous papules with central crusted ulcers. This patient was diagnosed HIV since 2017 and he received ART with Bictegravir/Emtricitabine/Tenofovir Alafenamide with poor adherence. The patient had a low CD4 count of 19 cells/mm^3^ and a high HIV viral load of 81,500 copies/mL upon admission to the hospital. The patient had been in his usual state of health until one week prior to admission, when he began to experience pain in the occipital area and the neck. A brain CT was arranged to rule out mass lesion, herniation, and other structural abnormalities. Following admission, *Cryptococcus neoformans* meningitis with fungemia was diagnosed with CSF analysis and serum laboratory survey. In CSF, it revealed a positive India ink result, latex of Cryptococcus test (+, 1:512) (IMMY CrAg^®^ LFA), and *Cryptococcus neoformans* in CSF culture. In serum analysis, positive latex of Cryptococcus test (1:4096) (IMMY CrAg^®^ LFA) and *Cryptococcus neoformans* in blood culture were found. 

**Figure 1:**
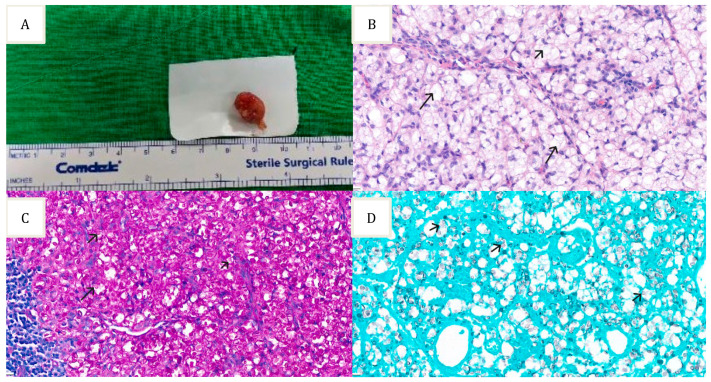
Specimen of the inguinal lymph node: (**A**) Left inguinal lymph node excisional biopsy; (**B**) Hematoxylin and eosin stain showing lymph node tissue with numerous fungal elements; (**C**) Periodic-Acid Schiff stain highlighting the fungal elements; (**D**) Gomori’s Methenamine Silver stain highlight the fungal elements.

We started amphotericin B and flucytosine upon admission for the treatment of cryptococcal meningitis. However, we discontinued flucytosine on day 19 due to severe neutropenia (serum WBC decreased from 6460/μL to 1320/μL) and anemia (Serum Hb from 11.8 g/dL to 6.8 g/dL). Amphotericin B was switched to liposomal amphotericin B due to worsening renal function (serum creatinine increased from 1.04 mg/dL to 1.53 mg/dL). Although symptoms presented upon admission had been alleviated, bilateral inguinal lymphadenopathy persisted following antifungal therapy. These lymph nodes were painless, fixed, and 1 cm in diameter. An excisional biopsy was performed on the inguinal lymph nodes, which revealed a fungal infection caused by *Talaromyces amestolkiae*, instead of *Cryptococcus neoformans* (Figure 1).

To further verify the presence of *T. amestolkiae*, we sent the lymph node specimen to Tri-ibiotech, Taiwan. This company performed amplification and sequencing of the internal transcribed spacer rDNA area (ITS) and β-tubulin (BenA) with primer sets ITS5/ITS4 and Bt2a/Bt2b [5]. ITS works as a DNA sequencing marker for fungi, but there is insufficient variation to distinguish all the closely related species of *Penicillium* and many other genera of ascomycetes. Because the ITS reference sequence has its limitations, a second DNA sequencing marker is needed to identify isolates to species level. BenA is the best option for a secondary identification marker for *Penicillium*. The ITS sequence of our strain showed 98.8% identity to *T. amestolkiae* strain M26 (MN086355.1), while the BenA sequence was identical (99.29%) to that of the other strain *T. amestolkiae* isolate AC5806 (KJ413360.1). As suggested, we further analyzed the ITS and BenA sequences of M26 and AC5806. The length of these two sequences was different as the M26 ITS (MN086355.1) was 868 bp and the AC5806 ITS (KJ413385.1) sequence was 536 bp in length. The overlapping region was from 115 to 650 bp of M26 ITS and was 100% identical. The BLAST of our ITS showed the overlapping region was from 119 to 701 of M26 ITS. Regarding to the BenA sequence of these two referenced strains, the M26 BenA sequence (MN097073.1) was 428 bp and the AC5806 BenA sequence was 422 bp in length, and the identity of these two sequences was 99% (400/403). In conclusion, the ITS and BenA sequences of our sample showed that there was a *T. amestolkiae* in the patient. 

Antiretroviral therapy with abacavir, lamivudine, and dolutegravir was initiated after 6 weeks of anti-fungal medications. India ink staining of the cerebrospinal fluid was negative after 4 weeks of amphotericin B liposome during this admission. We changed amphotericin B to voriconazole as maintenance therapy for cryptococcal meningitis and the *T. amestolkiae* infection. The patient was discharged under stable condition.

## 3. Results and Discussion

This report described an AIDS patient with cryptococcal meningitis who also had a *Talaromyces amestolkiae* infection, which presented with lymphadenopathy. It was difficult to diagnose this fungal infection due to the limitation of clinical diagnostic tools and challenges in distinguishing *T. amestolkiae* from other *Talaromyces* species without performing PCR or genome sequencing. A limited number of *T. amestolkiae* infections have been reported to date, and thus, this species is still relatively unknown. Nevertheless, the advent of PCR assays and DNA sequencing technology has improved its identification and raised awareness of its existence. The use of PCR combined with microscopic examination, fungal culture, and clinical symptoms allows for more accurate diagnosis of *T. amestolkiae*. When dealing with uncommon pathogenic fungi, misdiagnosis or failure to identify the correct pathogen is common. Therefore, we reviewed the reported pathogenic species of *Talaromyces* and the differences between *T. marneffei* and *T. amestolkiae*. 

In 2011, Samson et al. proposed that *Penicillium* subgenus *Biverticillium* should be taxonomically unified with the *Talaromyces* species. Using sequencing data from the ITS and RPB loci, they determined that it was a phylogenetically distinct species characterized by conidiogenous cells with acerose and/or loosely woven ascocarp walls and placed them in the *Talaromyces* genus [6]. *Talaromyces* species may easily confuse the clinician due to ambiguous microscopic findings. Hence, early diagnosis with DNA sequencing markers is necessary due to the high mortality rate of untreated *T. marneffei* patients. With this goal in mind, sequencing data from the ITS, BenA, and RPB2 gene regions were used to define relationships within *Talaromyces* [2]. There are other possible secondary markers, including calmodulin (CaM) and the second largest subunit of RNA polymerase II (RPB2) genes [7]. The ITS reference sequence of *T. marneffei* is JN899344 (alternative markers: BenA = JX091389; CaM = KF741958; RPB2 = KM023283). The ITS reference sequence of *T. amestolkiae* is JX315660 (alternative markers: BenA = JX315623; CaM = KF741937; RPB2 = JX315698) [2]. With these DNA sequencing markers, we can accurately identify fungal pathogens and prescribe corresponding treatment precisely.

Several clinically significant *Talaromyces* species listed in Table 1 were previously reported. Among these species, *T. marneffei* is a fatal fungal pathogen that poses a particular risk to immunocompromised patients. This species was previously known as *Penicillium marneffei*; cases infected with this pathogen were mainly distributed in East Asian countries, such as China, Taiwan, Thailand, and Vietnam [8,9,10,11]. *Talaromyces indigoticus* was isolated from skin and nail lesions from a man who was affected by onychomycosis in Western Panama [12]. *Talaromyces piceus* was found in an X-linked chronic granulomatous disease (X-CGD) patient who suffered from rib osteomyelitis [13]. *T. piceus* fungemia was diagnosed in a woman with alcohol-abuse disorder who was diagnosed cholangiocarcinoma after autopsy [14]. *T. amestolkiae* and *Talaromyces stollii* were isolated from the lungs and sputum of immunocompromised patients [4]. In this report, *T. amestolkiae* was isolated from the lymph node of our AIDS patient, suggesting a new clinical presentation.

As *T. amestolkiae* has not been encountered as frequently compared to *T. marneffei*, we searched for relevant articles in medical databases and compared the two species with respect to risk factors, clinical manifestations, microbiological diagnosis, and treatment regimen to aid in future clinical diagnosis and treatment. A summary of the information is presented in Table 2.

Both *T. amestolkiae* and *T. marneffei* were predominantly isolated from immunocompromised patients. *T. amestolkiae* was reported in sputum samples from cystic fibrosis patients, bronchoalveolar lavage fluids from patients with acute lymphoblastic leukemia who recently underwent chemotherapy, and a lymph node biopsy from our AIDS patient with a CD4 count of 19 cells/mm^3^. Furthermore, *T. marneffei* was found in patients with a range of conditions, including AIDS, autoimmune diseases, cancers, and diabetes mellitus.

The patient with *T. amestolkiae* pneumonia exhibited symptoms of productive cough, dyspnea, and low-grade fever, while our patient presented with painless inguinal lymphadenopathy. In addition, *T. marneffei* infection typically resulted in subacute non-specific symptoms, such as fever, weight loss, hepatosplenomegaly, lymphadenopathy, and respiratory and gastrointestinal abnormalities. Furthermore, *T. marneffei* induced central necrotic papules over the face, chest, and extremities; the appearance of the papules resembles molluscum contagiosum. A definite diagnosis of *Talaromyces* relies on fungal culture and DNA sequencing. The culture of *T. amestolkiae* has center-raising and low margin colonies and grows rapidly on Czapek yeast agar (CYA) and malt extract agar (MEA). The culture has red soluble pigment and dark to violet-brown reverses. However, these features were absent in some strains [2]. Thus, it can easily be confused with other *Talaromyces* species such as *T. purpurogenus*. On the other hand, *T. marneffei* is the only dimorphic species in the *Talaromyces* genus. It produces filamentous growth at 25 degrees Celsius and a yeast phase at 37 degrees Celsius. Red soluble pigment was found on general media and conidiophores have flask-shaped to acerose phialides [2]. Because both fungi share similar growth characteristics on culture media, they can be difficult to distinguish by culture alone.

The antifungal therapy for *T. amestolkiae* was uncertain and there was a patient who had *T. amestolkiae* pulmonary infection. In the case report, the administration of voriconazole was initiated for the patient based on the suspicion of aspergillosis. Because the clinical improvement of the patient, they continued the voriconazole and it showed resolved symptoms and radiographic improvement after two months [15]. As for our patient who presented *T. amestolkiae* lymphadenopathy and cryptococcal meningitis, he received amphotericin B for 6 weeks then oral voriconazole.

For *T. marneffei* infection, the treatment includes three phases: induction, consolidation, and maintenance. The recommended induction therapy for all patients is intravenous amphotericin B for 2 weeks [22]. The induction phase is followed by consolidation therapy with oral itraconazole 200 mg every 12 h for 10 weeks [26]. After receiving the treatment of induction and consolidation, the patient was suggested to continue the maintenance therapy (secondary prophylaxis) with oral itraconazole 200 mg/day until the CD4 count rises above 100 cells/mm^3^ for more than 6 months [27]. In the event that the patient experiences intolerance to amphotericin B therapy, voriconazole could be considered as an alternative treatment option. In the subsequent consolidation phase, voriconazole can be continued or itraconazole may be considered as suitable therapeutic alternatives [28,29].

When HIV-infected patients are diagnosed with cryptococcal meningitis, clinicians tend to attribute all of the clinical manifestations to a single pathogen. However, in our case, we discovered through further investigations that the patient was simultaneously infected with both *C. neoformans* and *T. amestolkiae*. In addition to the two species found in our case, previous studies disclosed other co-infectants were *Mycobacterium*, *Pneumocystis jiroveci*, *Histoplasma*, *T. marneffei*, and *Aspergillus*. Details related to these events are presented in Table 3. These pathogens mainly caused lung infections, lymphadenopathy, and skin lesions. Each patient received treatments for Cryptococcal infection, except for one with non-tuberculous mycobacteria infection, since the report was issued after the patient had been discharged. Most patients completed treatments and were discharged with oral medications. Diagnosing co-infections with fungal pathogens can be challenging. Hence, clinicians should maintain a heightened level of attentiveness and employ optimal diagnostics to identify possible co-pathogens.

## 4. Conclusions

To our knowledge, this is the first documented instance of concurrent infection with both *C. neoformans* and *T. amestolkiae* in a patient with AIDS. These findings highlight the possibility of co-infections with fungal pathogens in immunocompromised patients and underscore the importance of maintaining a high level of clinical suspicion in these cases. Immunocompromised patients have a high risk of getting fungal infections. Broad-spectrum antifungals are frequently prescribed empirically without definite microbiological diagnosis, which may result in the development of drug resistance. Clinicians should be vigilant in utilizing appropriate diagnostic tools to identify the causative agents, which can guide therapeutic strategies. This report further illustrates that *T. amestolkiae* is not only an environmental fungus but also a human pathogen which morphologically resembles *Penicillium*. In cases where conventional diagnostic methods fail to differentiate closely related fungi, DNA sequencing can be utilized for further verification. The administration of amphotericin B followed by voriconazole successfully treated our patient. However, due to the limited number of cases reported, further studies are required to determine the optimal treatment for *T. amestolkiae*. The continued surveillance of fungal infections in immunocompromised patients is essential for identifying and managing co-infections and developing effective treatment strategies.

## Figures and Tables

**Table 1:** Clinically relevant *Talaromyces* species.

Species	Country	Underlying Conditions	Treatment	Outcome
*T. amestolkiae*	New Caledonia [4] Taiwan	AML case AIDS patients Cystic fibrosis case ALL case	Not mentioned in article	Not mentioned in article
*T. marneffei*	East Asian countries including China, Taiwan, Thailand, and Vietnam	AIDS	Amphotericin B then Itraconazole	Mortality rate: ~75% in untreated patient ~25% in received treatment patient
*T. indigoticus*	Nepal, Japan, Thailand, and Western Panama [12]	Diabetes mellitus	Not mentioned in article	Not mentioned in article
*T. piceus*	Argentina [13]	X-linked chronic granulomatous disease (X-CGD) patient	Amphotericin B then voriconazole due to renal impairment Surgical resection of affected ribs	Recovered and free from fungal infections
	Germany [14]	Alcohol-abused and cholangiocarcinoma		Deceased before treatment
*T. stollii*	France, Hamburg, and Netherlands [4]	lung transplantation, France AIDS patient; the Netherlands	Not mentioned in article	Not mentioned in article

**Table 2:** Comparison of *T.amestolkiae* and *T.marneffei*.

	*Talaromyces amestolkiae*	*Talaromyces marneffei*
Risk factors	Immunocompromised patient. Case reported in an acute lymphoblastic leukemia patient [15] and we found this pathogen in an AIDS patient.	AIDS (majority of cases having a CD4 count <100 cells/mm^3^) [16,17,18], autoimmune disorders, cancer, diabetes mellitus [19,20,21].
Clinical manifestations	Respiratory symptoms Productive cough, mild dyspnea, and occasional low grade fever [15] Other symptoms Lymphadenopathy	Respiratory symptoms Nonproductive cough, fever, dyspnea, and chest pain Gastrointestinal symptoms Diarrhea and abdominal pain Skin lesions Papules on the face, chest, and extremities. Subsequently, the center of the papule becomes necrotic, giving the appearance of an umbilicated papule, which can resemble molluscum contagiosum [22]. Mucosal lesions Mucosal lesions appear similar to skin lesions. Distributed in the oral cavity, oropharynx, hypopharynx, stomach, colon, and genitalia had been reported [9,23,24,25]. Other symptoms Weight loss, hepatomegaly, splenomegaly, and/or generalized lymphadenopathy [22].
Definitive diagnosis		
Culture	7 days in CYA at 25 °C [2]	May need 4~7 days to grow
	- Colonies slightly raised at the center, slightly sulcate; margins low, plane, entire (2–3 mm) - Soluble pigments very weak brownish-red, in some strains absent (at 37 °C yellowish-red) - No exudates	- mold-to-yeast conversion (the only thermally dimorphic *Talaromyces* species) - grows readily in Sabouraud dextrose agar
	7 days in MEA at 25 °C	At 25 °C to 30 °C, yellow-green colonies with sulcate folds and a red diffusible pigment in the medium are produced. At 32 to 37 °C (yeast phase)
	- Colonies slightly raised at the center, plane, in some colonies black sclerotia produced in a longer cubation; margins low, plane, entire (3–5 mm) [2]	Morphological transition from a mold to a yeast, producing colonies without a red diffusible pigment [22]
Molecular diagnostics	PCR amplification Sequence identification of specific regions	PCR amplification Sequence identification of specific regions
Treatment		
	Pulmonary infection [15] Oral Voriconazole 200 mg every 12 h for 2 months Our patient (co-infected with cryptococcus neoformans meningitis) Amphotericin B for 6 weeks then oral Voriconazole	Recommended induction therapy [22] Amphotericin B, preferably liposomal amphotericin B 3 to 5 mg/kg body weight/day or Deoxycholate amphotericin B 0.7 mg/kg body weight/day, IV for 2 weeks
		Consolidation therapy oral itraconazole, 200 mg every 12 h for a subsequent duration of 10 weeks [26]
		Maintenance therapy (or secondary prophylaxis) oral Itraconazole 200 mg/day
		- Prevent recurrence until the CD4 count increases above 100 cells/mm^3^ for 6 months [27]
Special consideration		For patients who cannot tolerate any form of amphotericin induction therapy with IV Voriconazole 6 mg/kg every 12 h on day 1 (loading dose), then 4 mg/kg every 12 h or with oral Voriconazole 600 mg every 12 h on day 1 (loading dose), then 400 mg every 12 h for 2 weeks is recommended [28,29]

**Table 3:** Co-infections with *Cryptococcus*.

Pathogens	Underlying Disease	Specimen of Co-Infectants	Specimens of *Cryptococcus*	Treatment Strategy	Treatment for *Cryptococcus*	Treatment for Co-Infectants	Outcome
*Pneumocystis jiroveci* [30]	AIDS	* BAL ^1^	BAL ^2^ and Blood ^2^	*Pneumocystis jiroveci,* then ART, then asymptomatic *cryptococcus*	Voriconazole	Cotrimoxazol	Complete resolution of the cavitation
Non-tuberculous *Mycobacteria* [31]	AIDS, syphilis	Sputum/BAL ^1^ (CMV, EBV, *Candida albicans* also detected)	Blood ^2^	Treat NTM only because the culture results are later than the patient’s discharge	No treatment	Trimethoprim-sulfamethoxazole and steroids	Not mentioned
*Mycobacterium avium complex* [32]	AIDS	Lymph node biopsy ^2^	Blood ^1^ and * CSF ^1^	Treat *Cryptococcus* first, then ART, then MAC	Amphotericin B and flucytosine then fluconazole	Azithromycin, ethambutol and rifabutin	Good clinical evolution
Histoplasmosis [33]	AIDS	Lymph node biopsy	Blood and Sputum	HARRT then treat *Cryptococcus* infection	Amphotericin B and flucytosine then fluconazole		Continue * ART and Fluconazole
*T. marneffei*	AIDS [34]	Skin papules culture	* CSF	HARRT and treat *Cryptococcus* infection	Amphotericin B then itraconazole	Not mentioned	Skin papule disappeared and Continue * HAART therapy
	Hemolytic anemia with 8-year steroid history [35]	Blood ^1^ and lymph node aspiration	Blood culture ^2^	Voriconazole for *T. marneffei* and *Cryptoccocus*	Voriconazole	Voriconazole	Discharged with oral voriconazole
*Aspergillus* [36]	Multiple myeloma	* BAL ^2^	Pulmonary infection ^1^, not mentioned about the specimen	Treat *Cryptococcus* first then *Aspergillus*	Amphotericin B and flucytosine, then fluconazole	Fluconazole was shifted to voriconazole for additional coverage	Discharged with oral voriconazole
*Mycobacterium tuberculosis* [37]	No remarkable history	Transbronchial biopsy specimen from RUL lung and Sputum ^1^	* BAL ^1^ & CSF ^2^	combination of anti-TB and antifungal therapy	Amphotericin B and flucytosine then fluconazole	Isoniazid+ rifampin+pyrazinamide+ ethambutol	Hold fluconaconazole for nephrotoxicity; Discharged
*Mycobacterium abscessus* [38]	Lupus nephritis and 10-year corticosteroid history	Sputum	Sputum	Patient refused inpatient care	Itraconazole	Clarithromycin and faropenem	No recurrence was observed

* BAL: Bronchial-alveolar lavage; CSF: Cerebrospinal fluid; HAART: Highly Active Anti-Retroviral Therapy; ART: Anti-Retroviral Therapy. ^1^: Specimens at initial diagnosis. ^2^: Specimens with subsequent pathogen detection.

## Data Availability

Not applicable.
